# Update on the diagnosis and treatment of neuromyelitis optica: Recommendations of the Neuromyelitis Optica Study Group (NEMOS)

**DOI:** 10.1007/s00415-013-7169-7

**Published:** 2013-11-23

**Authors:** Corinna Trebst, Sven Jarius, Achim Berthele, Friedemann Paul, Sven Schippling, Brigitte Wildemann, Nadja Borisow, Ingo Kleiter, Orhan Aktas, Tania Kümpfel, Neuromyelitis Optica Study Group (NEMOS)

**Affiliations:** 1 Department of Neurology, Hannover Medical School, Hannover, Germany; 2 Division of Molecular Neuroimmunology, Department of Neurology, University of Heidelberg, Heidelberg, Germany; 3 Department of Neurology, Klinikum rechts der Isar, Technische Universität München, Munich, Germany; 4 Clinical and Experimental Multiple Sclerosis Research Center, Department of Neurology, Charité-Universitätsmedizin Berlin, Berlin, Germany; 5 NeuroCure Clinical Research Center and Experimental and Clinical Research Center, Charité-Universitätsmedizin Berlin and Max Delbrück Center for Molecular Medicine, Berlin, Germany; 6 Neuroimmunology and Multiple Sclerosis Research Section, Department of Neurology, University Hospital Zürich, Zürich, Switzerland; 7 Department of Neurology, St. Josef-Hospital, Ruhr-Universität, Bochum, Germany; 8 Department of Neurology, Medical Faculty, Heinrich-Heine-Universität Düsseldorf, Düsseldorf, Germany; 9 Institute of Clinical Neuroimmunology, Medical Campus Grosshadern, Ludwig-Maximilians-Universität, Munich, Germany

**Keywords:** Neuromyelitis optica, Differential diagnosis, Diagnostic tests, Therapy

## Abstract

Neuromyelitis optica (NMO, Devic’s syndrome), long considered a clinical variant of multiple sclerosis, is now regarded as a distinct disease entity. Major progress has been made in the diagnosis and treatment of NMO since aquaporin-4 antibodies (AQP4-Ab; also termed NMO-IgG) were first described in 2004. In this review, the Neuromyelitis Optica Study Group (NEMOS) summarizes recently obtained knowledge on NMO and highlights new developments in its diagnosis and treatment, based on current guidelines, the published literature and expert discussion at regular NEMOS meetings. Testing of AQP4-Ab is essential and is the most important test in the diagnostic work-up of suspected NMO, and helps to distinguish NMO from other autoimmune diseases. Furthermore, AQP4-Ab testing has expanded our knowledge of the clinical presentation of NMO spectrum disorders (NMOSD). In addition, imaging techniques, particularly magnetic resonance imaging of the brain and spinal cord, are obligatory in the diagnostic workup. It is important to note that brain lesions in NMO and NMOSD are not uncommon, do not rule out the diagnosis, and show characteristic patterns. Other imaging modalities such as optical coherence tomography are proposed as useful tools in the assessment of retinal damage. Therapy of NMO should be initiated early. Azathioprine and rituximab are suggested as first-line treatments, the latter being increasingly regarded as an established therapy with long-term efficacy and an acceptable safety profile in NMO patients. Other immunosuppressive drugs, such as methotrexate, mycophenolate mofetil and mitoxantrone, are recommended as second-line treatments. Promising new therapies are emerging in the form of anti-IL6 receptor, anti-complement or anti-AQP4-Ab biologicals.

## NEMOS

The Neuromyelitis Optica Study Group (NEMOS; see http://www.nemos-net.de) was initiated in 2008 by neurologists at 25 German university and academic teaching hospitals as an open-access network to improve the care of patients with neuromyelitis optica (NMO). Since then, the group has organized a number of national and international meetings and symposia on NMO, collected and analyzed data on epidemiological, clinical, and magnetic resonance imaging (MRI) characteristics of NMO in a large German cohort [1], and published recommendations on the diagnosis and treatment of NMO in Germany [2]. In the following report, these recommendations are updated to reflect the most recent literature in the field and current scientific knowledge. The 2010 guidelines of the European Federation of Neurological Societies (EFNS) on the diagnosis and management of NMO, guidelines published by an international expert group [3], and the evidence-based guidelines on clinical evaluation and treatment of transverse myelitis published by the Therapeutics and Technology Assessment Subcommittee of the American Academy of Neurology can also be referred to for additional information [4, 5].

## Introduction

Neuromyelitis optica is an immune-mediated chronic inflammatory disease of the central nervous system (CNS) [1, 6, 7]. NMO was first described in the 19th century and was long considered a clinical variant of multiple sclerosis (MS) [8–13]. Clinically, it presents with optic neuritis (ON) and myelitis, often characterized by poor or no recovery. Imaging typically shows longitudinally extensive lesions spanning three or more vertebral segments. Histopathologically, NMO is characterized by astrocytic damage, demyelination, neuronal loss, and often pronounced necrosis [14–16]. The discovery of perivascular antibody and complement deposition within active lesions and the subsequent discovery of specific autoantibodies (aquaporin-4 antibodies, AQP4-Ab; also termed NMO-IgG) in the serum of NMO patients indicated that humoral immunity is involved in the majority of cases. AQP4-Ab-positive NMO is now distinguished from MS as an independent disease entity [17–27]. Accordingly, serological identification of NMO-IgG has also been included as an additional criterion in all diagnostic criteria for NMO currently in use [2, 4, 28, 29].

## Epidemiology

Solid data on the incidence and prevalence of NMO are lacking. Its prevalence is estimated to range from less than 1 to 4.4/100.000 in the Western world [31–33]. In the past, many patients (>20 %) with NMO were misdiagnosed with MS, especially before NMO-IgG testing became widely available [1]. Notably more women than men have NMO (ratio 9:1, compared with just 2:1 in MS) [1, 34]. The median age at onset, 39 years, is approximately 10 years higher than in MS [1, 30]. However, cases of onset during childhood and in the elderly have been described [1, 35–38]. NMO takes either a relapsing or a monophasic course, with the former predominating (approximately 80–90 % of cases) [1, 30]. Compared with MS, AQP4-Ab-positive NMO is more frequently associated with other autoimmune diseases such as myasthenia gravis, systemic lupus erythematosus, Sjögren’s syndrome, celiac disease, and sarcoidosis [1, 39–52]. In up to 20–30 % of cases, NMO attacks are preceded by infection or vaccination [1, 7]. Age at onset and genetic factors may influence the clinical outcome [53].

Only few reports on the influence of pregnancies in NMO exist. Two studies reported an increase in relapse rate in the first 3 or 6 months, respectively, post partum [54, 55].

## Diagnostic criteria

According to the criteria proposed by Wingerchuk et al. [28] in 2006, a diagnosis of NMO can be made with high specificity if, in addition to a history of at least one episode of ON and one episode of myelitis, two of the following three supporting criteria are met:

1. Contiguous spinal cord MRI lesion extending over three or more vertebral segments
2. Brain MRI not meeting Paty’s diagnostic criteria for MS^1^ [56] at disease onset.^2^
3. NMO-IgG seropositive status^3^

Of note, the sensitivity and specificity of these criteria [28] were defined using brain MRI at disease onset as first preference. If the first scan available was taken at a later time and was *negative* for MS, it was assumed that the onset scan would also have been negative. By contrast, the authors did not indicate whether the brain MRI criterion should be applied at all if the first available scan was taken at a later time and *met* MS criteria. However, we believe that the diagnostic criteria proposed by Wingerchuk et al. should, in general, not be applied to rule out NMO if any of the paraclinical procedures required to evaluate the three supporting criteria were not performed. Of course, a diagnosis of NMO can be made if the index events and any two of the three supporting criteria are met, even though information on the third supporting criterion is not available.

More broadly, those criteria should be primarily used to make, rather than to exclude, a diagnosis of NMO, because brain lesions and (far more rarely) short spinal cord lesions—individually or combined—may in fact be present in patients with otherwise typical NMO (as confirmed by AQP4-Ab seropositivity and/or occurrence of longitudinal extensive transverse myelitis (LETM) in the later disease course in these patients) [1].

## “NMO-spectrum disorder”—abortive and atypical manifestations

AQP4-Ab have been demonstrated in patients with conditions other than classical NMO, including isolated LETM, as defined by lesions spanning over more than three segments, monophasic or recurrent isolated ON, and certain types of brainstem encephalitis (particularly if the diencephalon or the medulla oblongata is involved) [57–59]. Brainstem manifestations frequently include intractable hiccups or vomiting, symptomatic narcolepsy, and neuroendocrine dysfunctions [58–60], and may also precede ON or myelitis [1, 61–63]. It has been suggested that posterior reversible encephalopathy syndrome might also present in the context of NMO [64]. Recently, olfactory dysfunction has been described in patients with NMO [65]. Whether AQP4-Ab causes damage outside the CNS (e.g., placenta [1–3], stomach [4], muscle [5, 6], or inner ear [7]) is currently under investigation.

In children, an even broader spectrum of encephalitic manifestations has been described, in particular regarding seizures [36–38]. In a German cohort, 152 of 175 patients (87 %) did not present at disease onset with simultaneous myelitis and bilateral ON, but with isolated (mostly unilateral) ON, isolated myelitis, or brainstem encephalitis. Similarly, 89 of 106 patients (84 %) presented with abortive or atypical symptoms in a British-Japanese cohort [1, 53]. As most of these patients later developed NMO, various groups have suggested classifying these symptoms—if occurring in the context of AQP4-Ab seropositivity—as ‘high-risk syndromes for NMO’ (HRS) and referring to AQP4-Ab-positive classical NMO and AQP4-Ab-positive HRS as ‘NMO spectrum disorder’ (NMOSD) or ‘autoimmune AQP4 channelopathy’ [74–77]. The inconsistent use of the term ‘NMOSD’ has recently been criticized [8].

## Clinical evaluation when NMO is suspected

### Medical history and physical examination

A detailed medical history is essential. The neurological and physical examination should focus not only on the primary symptoms, but also on disease indicators that could suggest alternative diagnoses or concomitant autoimmune disorders, which are frequently present in patients with AQP4-Ab-positive NMO [1, 45, 47]. Special attention should be paid to brainstem symptoms, neuropathic pain, and painful tonic spasm [78], which have been shown to occur more frequently in NMO than in MS, and which have a demonstrated serious impact on quality of life [1, 58–63, 79, 80].

### Basic laboratory tests

The following tests are recommended for exclusion of differential diagnoses or confirmation of NMO-associated diseases: differential blood count, coagulation, serum chemistry, blood sedimentation, blood glucose, vitamin B12 [81], folic acid, antibodies associated with connective disorders (ANA/ENA, anti-ds-DNA antibodies, lupus anticoagulant, antiphospholipid antibodies, ANCA, etc. [45]), urine analysis and sediment, *Treponema pallidum* hemagglutination assay, and paraneoplastic antibodies (in particular, anti-CV2/CRMP5 [82] and anti-Hu). Based on clinical presentation and cerebrospinal fluid (CSF) results, analysis for copper deficiency (to exclude it as a cause of myelopathy) and zinc poisoning (if suspected) should be performed [83]. Moreover, recently, antibodies to myelin oligodendrocyte glycoprotein (MOG) have been reported in a subset of both adult and pediatric patients with (mostly AQP4-Ab-negative) NMO [84–86]; however, the exact diagnostic and therapeutic relevance of this finding is currently investigated [87].

### Detection of AQP4 antibodies

Several techniques are currently available to test for serum AQP4-Ab and can be categorized according to whether they are tissue-, cell-, or protein-based [18, 25, 88–97]. Using these serological tests, AQP4-Ab are detected in 60–90 % of patients who meet the clinical and radiologic criteria for NMO. The specificity of these assays varies between ~90 and 100 %. So-called cell-based assays using HEK293 cells transfected with recombinant, full-length human AQP4 have shown higher sensitivity and specificity than indirect immunofluorescence (IHC) [88, 90, 95, 98], enzyme-linked immunosorbent assays [95], and, in particular, radioimmunoprecipitation assays [93]. The prevalence of AQP4-Ab seems to be higher in female patients and in patients with relapsing disease [1, 99]. AQP4-Ab serum levels have been shown to be higher during relapse than during remission [88, 95, 96, 98, 100, 101]. However, levels during relapse vary considerably both inter- and intraindividually, with no apparent threshold for relapse induction [100, 102]. AQP4-Ab remain detectable in many cases during immunosuppressive treatment (with the exception of plasma exchange), as long as sufficiently sensitive assays are used [100]. Whenever possible, however, AQP4-Ab testing should be performed on samples taken prior to treatment commencement [100]. Re-testing initially seronegative patients during an acute attack or a treatment-free interval may be advisable [38]. Routine testing of AQP4-IgM is currently not recommended [103]. The diagnostic value of AQP4-Ab in the CSF remains controversial [104, 105]. AQP4-IgG are relatively stable over a period of at least a week at room temperature or 4 °C [106]; however, shipment on dry ice may be advisable for low-titer or CSF samples.

While AQP4-Ab are potentially of high diagnostic and therapeutic relevance, a critical need exists to challenge the current clinical practice of AQP4-Ab testing, for the following reasons: (1) Due to the low incidence of AQP4-IgG-positive NMO, the vast number of patients currently tested for AQP4-Ab [107], the limited specificity of some diagnostic assays, and the insufficient number of controls included in almost all past studies, the ratio of false-positive to true-positive test results might be higher than generally expected. This is even more problematic in patients presenting with a first episode of isolated ON or brainstem encephalitis, who are less frequently positive for AQP4-Ab. (2) Assays with insufficient sensitivity, such as IHC, have been broadly used in the past and are still partly in use. False-negative results may lead to treatment with interferon-beta or natalizumab for suspected MS; these two drugs are thought to cause disease exacerbation or to have no therapeutic benefit, respectively, in patients with NMO. On the other hand, false-positive results might prompt treatment with immunosuppressants with no established efficacy in MS and potentially serious side effects. Manufacturer-independent, multicenter comparative trials that include multiple assays as well as a sufficient number of adequate controls (≥1,000) are urgently required. Ideally, AQP4-Ab test results should therefore be confirmed using a second, methodologically independent assay with high sensitivity and specificity, and, in the case of conflicting results, a third assay. Moreover, repeat testing is recommended in equivocal cases.

### Cerebrospinal fluid diagnostics

Examination of CSF includes cell count, cytology, protein, lactate, albumin CSF/serum ratio, IgG, IgA, and IgM CSF/serum ratios, oligoclonal bands (OCB), and the MRZ (measles, rubella, and varicella zoster virus) reaction. Moderate pleocytosis (mostly lymphomonocytic) is often a feature of NMO, and can be more prominent than in MS, but usually less than in infectious myelitis [7, 108–111]. On the other hand white cell counts are normal in around 40 % of CSF samples during acute relapses in patients with AQP4-Ab positive NMO [111]. Neutrophil (sometimes also eosinophil) granulocytes are frequently detected and, especially if present along with elevated lactate levels, may lead to the incorrect diagnosis of infectious myelitis in individual patients [111–113]. OCBs are positive in approximately 30 % of cases [111]. Repeating the CSF analysis can be useful for individual cases, since—unlike in MS—most CSF alterations in NMO mainly present during acute events and disappear during remission [111]. Moreover, an initial finding of OCB positivity followed by OCB negativity later in the disease course is indicative of NMO [104, 111, 114], but not MS. Testing for a positive MRZ reaction (defined as intrathecal IgG synthesis against at least two of the three pathogens) can be useful for differentiating between NMO and MS, as it is frequently positive in MS but not in NMO [1, 115]. More recently, concentrations of interleukin-6 (IL-6) and of the soluble IL-6 receptor (sIL-6R) were found to be higher in the CSF of NMO patients than of MS patients, and these may prove to be useful markers for differentiating NMO from other demyelinating diseases [116–118]. Whether measurements of glial fibrillary acidic protein (GFAP) in serum and/or CSF are of differential diagnostic value in NMO remains to be clarified [119–124].

### Electrophysiology

Visual evoked potentials, median and tibial somatosensory evoked potentials, and motor evoked potentials should be performed. Visual evoked potentials are frequently altered in NMO [125, 126]. A recent study found prolonged P100 latencies in around 40 % and reduced amplitudes or missing potentials in around 25 % of patients [125].

## Imaging

### Magnetic resonance imaging

Magnetic resonance imaging is the most important imaging technique in the differential diagnosis of NMO. Imaging of the entire CNS (cranial and spinal cord MRI) should always be performed, regardless of the primary presenting clinical signs and symptoms. Contrast agents are obligatory, as are follow-up examinations. Predominantly central longitudinally spinal cord lesions, usually extending over three or more vertebral segments, are typical of NMO [127]. These often, but not always, show contrast enhancement for weeks up to months after the onset of symptoms. Enhancement can be patchy and inhomogeneous. Extensive, centrally located necrosis and cavitation have been reported [128]. However, treatment can induce a marked improvement and sometimes full recovery. The lesions can also resemble ischemic lesions in the anterior spinal artery territory [129] or local tumours [130]. Additional presence of cerebral lesions does not exclude a diagnosis of NMO. Cerebral T2-/FLAIR hyperintensities exist in up to 60 % of NMO patients, although they are often clinically silent, frequently not classically oval-shaped (as typically seen in MS), and typically not visible on T1-weighted images [131, 132]. In two recent studies, 58 % and 63 %, respectively, of patients with NMOSD showed brain lesions and, of these, 18 % and 27 %, respectively, were considered diagnostic of MS [1, 132]. Brain lesions are generally located close to the ventricles, in the diencephalon and hypothalamus. Two recent ultrahigh-field MRI studies showed that—as opposed to MS lesions—NMO lesions in the brain are not characterized by central veins and that cortical lesions were absent in NMO [133, 134]; however, extensive lesions and MS-like findings are possible [57, 135–142].

### Optical coherence tomography

Optical coherence tomography (OCT) is a rapid and non-invasive technique for imaging unmyelinated CNS axons within the retina (the so-called retinal nerve fiber layer, RNFL). Recent technical advances have facilitated the high-resolution depiction of deeper retinal layers such as the ganglion cell layer. OCT is an increasingly popular tool in neuroimmunological research. Damage (thinning) to the RNFL in MS patients with and without a history of ON has been demonstrated by numerous groups. The suitability of OCT as a means of measuring disease progression and as a response marker for neuroprotective therapies in MS and other neurological conditions is currently being investigated [143–150].

A single acute attack of ON causes more severe damage to the RNFL in NMO than in MS, reflecting the poorer visual outcome in NMO-associated ON [7, 151, 152]. While MS patients experience progressive reduction of the RNFL over time compared with healthy controls, accrual of RNFL loss in NMO seems to be related to clinical attacks [153–159]. Whether OCT may contribute to NMO differential diagnosis is currently under investigation [160].

## Therapy

A curative treatment for NMO does not exist to date. Instead, the main treatment goals are:

1. Remission and improvement of relapse-associated symptoms

2. Long-term stabilization of disease course by means of relapse prevention

3. Symptomatic therapy of residual symptoms

This review focuses on relapse therapy and intermittent long-term therapy. For symptomatic treatment recommendations, please see the reviews of MS treatment by de Sa et al. [161] and Samkoff and Goodmann [162], both published in 2011, as the symptomatic management of NMO is similar.

The rarity of NMO and its frequently severe disease course hamper the performance of prospective, randomized controlled trials evaluating treatment efficacy. The recommendations presented here are thus mainly based on case reports, retrospective case series, and a few prospective studies, all of which only meet evidence class III–IV. Accordingly, several areas of ambiguity exist. In the case of seronegative NMO, which more often takes a monophasic course [1], it remains unclear whether the treatment should be the same as that for seropositive NMO. Therefore, infectious, parainfectious, metabolic, or paraneoplastic causes must definitely be ruled out before considering immunosuppressive treatments for patients with seronegative NMO. Similarly, no treatment studies focusing on patients with limited or atypical forms of APQ4-Ab-positive NMO have yet been performed. Despite this, early initiation of long-term immunosuppressive therapy to delay a second relapse is recommended, because such patients have a high risk of relapse and conversion to typical NMO [53, 163]. In most recent case series and retrospective studies, the efficacy of the investigated therapies was found to be the same for patients with typical NMO and with AQP4-Ab-positive NMOSD. In light of this, relapse and intermittent treatment of APQ4-Ab-positive patients with limited forms of NMO should follow that of patients with typical NMO.

### Treatment of acute disease attacks

After standard neurological examination and the exclusion of infection, steroids are applied on five consecutive days with 1 g methylprednisolone (MP) per day i.v. in combination with a proton pump inhibitor and thrombosis prophylaxis [164]. In the case of a confirmed diagnosis of NMO, and depending on severity of the attack, an oral steroid tapering period should be considered.

If the patient’s condition does not sufficiently improve or the neurological symptoms worsen, therapeutic plasma exchange (TPE, five to seven cycles) can be performed [165–169]. Notably, TPE was effective both in seropositive and in seronegative patients with NMOSD in some studies [166, 170]. Early initiation of TPE might be associated with better clinical outcome [168, 171, 172]. In some cases, e.g., if contraindications for TPE exist, a second course of steroids can be applied at a higher dosage of up to five times 2 g MP [173, 174]. In a retrospective review of 10 patients treated with intravenous immunoglobulins (IVIg) for acute relapses because of lack of response to steroids with/without TPE, improvement was noted in about 50 % of patients [175].

If the patient is known to have responded well to TPE during earlier attacks and the present attack is severe, TPE can also be considered as a first measure. Immunoadsorption is an option for patients with contraindication for TPE, such as hypersensitivity reactions, or if TPE is not available [176]; however, whether the treatment has the same therapeutic efficacy as TPE has not been investigated to date.

### Long-term treatment of NMO

As NMO takes a relapsing course in most cases, with often incomplete recovery and rapid accumulation of neurological deficits, long-term immunosuppressive treatment should be initiated once the diagnosis of NMO has been confirmed. This also applies to APQ4-Ab-negative NMO patients with a severe first relapse and incomplete remission. However, as seronegative NMO more often follows a monophasic course, it may be justified to taper immunosuppressive therapy after some years of disease stability and after careful assessment of the risks and benefits in this group of patients.

Data on the long-term treatment (>5 years) of NMO are sparse, all retrospective, and mainly concern azathioprine (AZA) and rituximab (RX). Accordingly, AZA and RX are currently the most widely used first-line therapies in NMO. No studies comparing the efficacy of these two therapies have been published.

The following section discusses the currently most widely used therapy regimens and reports on new and emerging NMO therapies.

#### Azathioprine

Several studies, including a large retrospective review of 99 patients with NMO/NMOSD, have shown AZA to reduce relapse rate and ameliorate neurological disability in NMO [100, 177, 178]. A dosage regimen of 2.5–3 mg/kg body weight/day orally with monitoring of hematologic parameters and liver enzymes is recommended. The lymphocyte count should decrease to between 600 and 1,000/μl with AZA therapy and the mean erythrocyte volume should increase by about 5 % from baseline [177]. If the treatment response is lacking or side effects present, the dose should be adjusted or, if necessary, a different treatment should be applied. As the treatment may only take full effect after 3–6 months, it should initially be combined with oral steroid therapy (1 mg/kg body weight/day), as oral steroids have been shown to suppress disease activity in NMO [14, 179]. Blood cell count and liver enzyme monitoring are mandatory. Thiopurine methyltransferase enzyme activity (TMTP) testing can be performed before AZA therapy, if available, since patients with low activity may be at higher risk for severe side effects [180].

#### Rituximab

B cell depletion with RX has been demonstrated as effective in the treatment of NMO in several clinical case series and retrospective analyses [100, 102, 181–185]. Although the patients in these studies generally had already received one or more previous treatments, RX is now increasingly also used in treatment-naïve NMO patients with high disease activity. Thus, RX is another option for first-line treatment in NMO/NMOSD and for patients who have not responded to previous immunosuppressive therapy (e.g., AZA).

RX treatment can be initiated using one of two different regimens: either two 1 g infusions of RX at an interval of 2 weeks or four weekly 375 mg/m^2^ body surface area (BSA) applications. To prevent infusion-related side effects, premedication (1 g paracetamol, 100 mg prednisolone, 4 mg dimethindene maleate i.v.) should be dispensed. Additionally, the infusion should be administered at a sufficiently slow speed and monitored. Increasing evidence shows that incomplete B-cell depletion and/or B-cell repopulation is associated with relapse risk in NMO [100, 102, 183, 186]. Because most patients remain B-cell deficient for 6 months after RX treatment, re-dosing every 6 months is considered to be an adequate retreatment frequency [183]. CD19/20-positive B cells and/or CD27+ memory cells may be used as surrogate markers for treatment monitoring and re-dosing [100, 102, 183, 185]. Whether long-term RX treatment at lower doses does in fact suppress disease activity, as suggested by first patient therapy cohorts and recent investigations [185–187], requires further investigation. Individual patients with NMO have been treated up to eleven times with RX without major side effects and with an acceptable safety profile. Cases of progressive multifocal leukoencephalopathy (PML) have been reported in patients with cancer and rheumatological diseases treated with RX, mostly in combination with other immunosuppressive therapies. To date, no incidents of progressive PML have been reported in NMO patients during RX therapy. However, more data on the efficacy and safety of RX treatment in NMO are required.

#### Mycophenolate mofetil

In a retrospective analysis of 24 patients, treatment with mycophenolate mofetil (MMF) (median dose 2,000 mg/day, ranging between 750 and 3,000 mg) was associated with a reduction in relapse frequency and stable or reduced disability in patients with NMOSD. Half the patients in the study had previously been treated with AZA [188]. The treatment effect occurs more rapidly for MMF than for AZA. In patients experiencing side effects or poor response to AZA, MMF is recommended as an alternative treatment. PML has not yet been observed in NMO patients during treatment with MMF, but has been encountered in transplant recipients [189].

#### Immunoglobulins

Individual case reports and a 2012 case series have shown that high-dose IVIg are potentially beneficial [190–192]. For example, a case series of eight Spanish NMO patients showed positive results using bimonthly IVIg treatment (0.7 g/kg body weight/day for 3 days) for up to 2 years [192]. Thus, IVIg therapy is suggested as an alternative for patients with contraindication to one of the other treatments or, particularly, in children.

#### Mitoxantrone

Two recent observational studies [193, 194] have reported a 75–80 % reduction in relapse rate during treatment with mitoxantrone (treatment duration up to 22 months), underlining prior reports on the efficacy of mitoxantrone in NMO. A dose of 12 mg/m² BSA of mitoxantrone was administered i.v. monthly for 3–6 months, followed by infusions of 6–12 mg/m² every 3 months. The maximum dose of mitoxantrone was 100–120 mg/m² BSA. Whether other regimes (e.g., sole quarterly infusions, frequently used in MS) are as efficacious is not known. Due to the side effects (cardiotoxicity, therapy-related acute leukemia [195–197]) and the limited duration of the therapy, we recommend mitoxantrone as a second-line therapy when the treatments described above fail or cannot be applied. As for MS, we recommend that the maximum cumulative dose should not exceed 100 mg/m^2^ BSA. In individual cases, treatment with up to 140 mg/m^2^ BSA can be administered by a physician experienced in the therapy, but only if a stringent risk–benefit analysis is performed and cardiac function is monitored throughout the treatment.

#### Cyclophosphamide

While preliminary evidence from a number of case studies (AQP4-Ab-positive and -negative; systemic lupus erythematosus- and Sjögren’s syndrome-associated; daily oral dose in one, IV pulse in six, immunoablative in one; combination with steroids, IVIg, or AZA in all) suggested a possible treatment response to cyclophosphamide (CYC) [100, 198–201], a recent retrospective analysis of seven Brazilian NMO cases (pulsed IV CYC) failed to show such effect [202]. In another study, three of four patients treated with pulsed IV CYC had to be switched to methotrexate later due to treatment failure [203]. In light of this, CYC is only recommended when other immunosuppressive therapies fail or are not available. The treatment may be applied in immunoablative doses (2,000 mg/day for 4 days) or at a dose of 600 mg/m^2^ BSA per administration (together with uromitexan). The dose should be adjusted according to changes in the total leukocyte count, and CYC should be applied only under the supervision of an experienced physician.

#### Interferon-beta/glatiramer acetate

Interferon (INF)-beta should not be used in patients with NMO, as several retrospective studies have shown that INF-beta treatment frequently results in NMO disease exacerbation [100, 204–208]. Glatiramer acetate has not been shown to have detrimental effects in NMO patients to date; with only three cases reported, however, insufficient data exist on glatiramer acetate as NMO treatment [100, 209, 210].

#### Methotrexate

In a retrospective study of 14 AQP4-Ab-positive patients, treatment with methotrexate, mainly prescribed as a second-line drug, was associated with a significant decrease in the median annualized relapse rate (ARR) and was relatively well-tolerated. After exclusion of relapses within the first 3 months of treatment or on subtherapeutic doses, the proportion of relapse-free patients was 64 %. Disability stabilized or improved in 79 % [211]. In 13 of 14 cases, however, concomitant immunosuppression with oral prednisolone (*n* = 11), rituximab (*n* = 1), or tacrolimus (*n* = 1) was applied, and the impact of this remains unclear. Treatment with methotrexate and prednisone also resulted in disease stabilization in a smaller and less well-documented retrospective case series (*n* = 7) [203], and in a pediatric patient on methotrexate monotherapy [37].

#### Natalizumab

The treatment of NMO with natalizumab should be avoided; a recent retrospective study reported clinical deterioration after natalizumab treatment in five NMO patients initially misdiagnosed with MS [212]. In line with this, Barnett et al. [213] and Jacob et al. [214] have also described natalizumab treatment as causing disease exacerbation in NMO patients.

#### Fingolimod

Min et al. [215] reported a patient who had been diagnosed with MS due to an MS-typical brain MRI (which met the criteria of Barkhof et al.), but without OCB and with a normal IgG index. The patient had been enrolled in a clinical trial with fingolimod after experiencing relapses during 2 years of INF-beta treatment. Clinical deterioration and increased MRI activity was found 2 weeks after initiation of fingolimod. Diagnosis re-evaluation showed anti-AQP4 antibodies, indicating NMOSD, and determined that the patient met the American–European Consensus Group Criteria (US-EU criteria) for Sjögren’s syndrome, based on anti-SSA antibody detection, a positive Schirmer’s test, and a lip biopsy with focal lymphocytic sialoadenitis.

#### Combination therapies

Combination therapy is a potential option for NMO patients who have a refractory course. Oral steroids combined with AZA led to a decrease in ARR in two more recent studies [177, 178]. Similarly, methotrexate in combination with oral steroids resulted in disease stabilization in two studies [203, 211]. Another recent study showed that cyclosporin A in combination with low-dose oral steroids is effective in NMO patients [216]. Methotrexate may be also combined with RX therapy as in rheumatoid arthritis. Individual case reports have also shown that intermittent plasmapheresis combined with immunosuppressive treatment reduces attacks of NMO [217].

#### Anti-IL-6 therapy and other new therapies

Recent reports have suggested that IL-6 plays a role in NMO, contributing to the persistence of NMO-IgG-producing plasmablasts in patients with NMO [218]. The hypothesis has been lent weight by studies showing a favorable effect of the IL-6 receptor-blocking antibody tocilizumab, already licensed for therapy of rheumatoid arthritis, in NMO patients who have failed to respond to other therapies [219–221]. Thus, tocilizumab may be another therapeutic option for such patients.

The monoclonal antibody eculizumab, which is directed against the complement component 5, showed considerable efficacy in a small, open-label study of 14 NMO/NMOSD patients with disease activity [222, 223]. Of the 14 treated patients, 12 remained relapse-free and two showed disease activity. Apart from meningococcal sepsis and sterile meningitis in one patient approximately 2 months after the first eculizumab infusion, no other drug-related serious adverse events were reported. However, confirmation from larger, phase III studies is needed; moreover, broad administration of eculizumab would be hampered by its presently exorbitant cost.

Recent experimental strategies, which showed some beneficial effect in animal models in vitro and in vivo, include the use of competitive, non-pathogenic AQP4-specific antibodies (e.g., aquaporumab) [224, 225], neutrophil elastase inhibitors [226], antihistamines with eosinophil-stabilizing actions [227], and enzymatic AQP4-IgG deglycosylation or cleavage [228, 229].

An isolated case report showing that autologous hematopoietic stem cell transplantation (AHSCT) failed to prevent further relapses in a NMO patient raised concerns about the treatment’s efficacy in NMO. However, an ongoing AHSCT trial involving 10 NMO patients is expected to shed light on whether some patients do benefit from the therapy [230]. Alemtuzumab, a B- and T-cell-depleting antibody previously used in MS trials with favorable outcome, did not show beneficial effects when used in individual NMO patients [220, 231].

### Summary for treatment recommendations

**Fig. 1:**
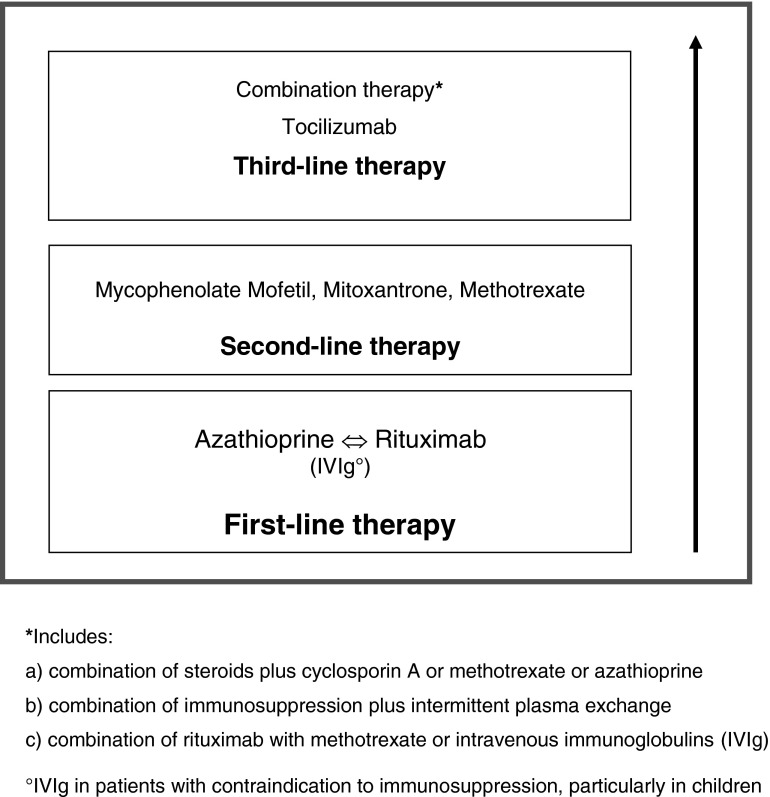
Long-term therapy of NMO

Based on the currently available evidence as summarized above, the NEMOS group gives the following treatment recommendations:

The frequently severe disease course of NMO calls for prompt initiation of immunosuppressive treatment once the diagnosis of NMO or AQP4-Ab-positive NMOSD has been confirmed, with azathioprine or rituximab as first-line treatment (see Fig. 1). In children or in patients with contraindications to immunosuppressive therapies, IVIg may be used as first-line therapy. In patients with NMOSD who are AQP4-Ab negative, therapy initiation depends on the severity and remission of the first relapse and the clinical course.

In the case of side effects or poor response, treatment can be switched from azathioprine to rituximab or vice versa, or to mycophenolate mofetil, methotrexate, or mitoxantrone. If disease progression occurs and if the above treatments fail, combination therapy or newer agents such as tocilizumab may be applied. Treatment with interferon-beta, natalizumab, and fingolimod should be avoided. How and whether treatment with the recently approved therapies for MS, teriflunomide and fumaric acid, influences the disease course in NMO patients remains to be elucidated.

In general, physicians must inform patients about the risks of side effects, such as malignancy, infertility, cytotoxicity and myelotoxicity, infections including PML, vaccination issues, and the need for contraception before initiating immunosuppressive therapies. Tests for pregnancy and chronic infections (HIV, hepatitis B and C) before treatment commencement are recommended.

## Future directions

The pathogenesis, diagnosis, and treatment of NMO are rapidly expanding research areas, as reflected by the steep increase in the number of publications on NMO since AQP4 antibodies were first described. Consequently, we expect major advances in all three areas over the next few years. Research on pathogenesis has progressed to studying the role of T-cells, neutrophils, eosinophils, and other cellular components of the immune system [27, 227, 232–234]. Several new potential therapeutic approaches have resulted from recent insights in NMO pathogenesis, including complement and neutrophil elastase inhibition [226] (eculizumab, sivelestat [235]), and the blocking of antibodies to AQP4 with monoclonal antibodies (aquaporumab), among others. The challenges in finding new and better medicines for NMO are the rareness of the disease and the unfavorable prognosis in many cases, which make clinical studies with placebo groups difficult. Although designing meaningful and clinically relevant NMO therapy studies is laborious, these trials will eventually increase our options for treating NMO.

## Members of the Neuromyelitis Optica Study Group (in alphabetical order)

P. Albrecht, Universität Düsseldorf; O. Aktas, Universität Düsseldorf; K. Angstwurm, Universität Regensburg; A. Berthele, Technische Universität München; F. Bischof, Zentrum für Neurologie Tübingen; N. Borisow, Charite Berlin; T. Böttcher, Bonhoeffer Klinikum Neubrandenburg; J. Brettschneider, Universität Ulm; M. Buttmann, Universitätsklinik Würzburg; B. Ettrich, Universität Leipzig; J. Faiss, Asklepios Klinik Teupitz; A. Gass, Universitätsklinikum Mannheim; C. Geis, Universitätsklinikum Jena; K. Guthke, Klinikum Görlitz; J. Havla, Institut für Klinische Neuroimmunologie LMU-München; H-P. Hartung, Universität Düsseldorf, K. Hellwig, Ruhr-Universität Bochum; B. Hemmer, Technische Universität München; F. Hoffmann, Krankenhaus Martha-Maria Halle; U. Hofstadt-van Oy, Klinikum Bayreuth; S. Jarius, Universität Heidelberg; P. Kern, Asklepios Klinik Teupitz; C. Kleinschnitz, Universitätsklinik Würzburg; I. Kleiter, Ruhr-Universität Bochum; W. Köhler, Fachkrankenhaus Hubertusburg; E. Kolesilova, Asklepios Klinik Teupitz; M. Krumbholz, Institut für Klinische Neuroimmunologie LMU-München; T. Kümpfel, Institut für Klinische Neuroimmunologie LMU-München; S. Langel, Landeskrankenhaus Rheinhessen; F. Lauda, Universität Ulm; M. Liebetrau, Agaplesion Ev. Bathildiskrankenhaus Bad Pyrmont; R. Linker, Universität Erlangen; W. Marouf, Heliosklinik Stralsund; M. Marziniak, Isar-Amper Klinik Ost München; I. Metz, Universität Göttingen; C. Mayer, Universität Frankfurt; A. Melms, Universität Erlangen; C. Münch, Charité Berlin; O. Neuhaus, Kreiskrankenhaus Sigmaringen; S. Niehaus, Klinikum Dortmund; F. Pache, Charité Berlin; F. Paul, Charité Berlin, H. Pellkofer, Universität Göttingen; R. Reuss, Bezirkskrankenhaus Bayreuth; A. Riedlinger, Asklepios Klinik Teupitz; M. Ringelstein, Universität Düsseldorf; S.P. Rommer, Universität Rostock; K. Ruprecht, Charité Berlin; S. Schippling, Universität Zürich (Schweiz); M. Schwab, Universität Jena; M. Stangel, Medizinische Hochschule Hannover, J. Stellmann, Universität Hamburg; F. Then-Bergh, Universität Leipzig; C. Trebst, Medizinische Hochschule Hannover; H. Tumani, Universität Ulm; C. Veauthier, Heliosklinik Stralsund; KP. Wandinger, Universitätsklinikum Schleswig–Holstein; R. Weissert, Universität Regensburg; B. Wildemann, Universität Heidelberg; C. Wilke, Nervenzentrum Potsdam; A. Winkelmann, Universität Rostock; L. Zeltner, Universität Tübingen; C. Zentner, Krankenhaus Martha Maria, Halle; U. Zettl, Universität Rostock; U. Ziemann, Universität Tübingen.

## Footnotes

1 Four or more white matter lesions, or more than three white matter lesions if one of these is located in the periventricular region.

2 If no cranial MRI was performed at disease onset, or the findings are unknown, the earliest available MRI should be used [28].

3 Detection of AQP4-Ab using recombinant methods can replace immunohistochemical detection of NMO-IgG [18], provided that the respective recombinant test has been demonstrated to yield equal or better sensitivity and specificity in clinically well-defined and sufficiently large patient and control collectives, *and* has been successfully validated using an NMO-IgG-positive patient collective. Of the testing systems described in the literature and currently available for diagnosis, mainly cell-based assays meet these requirements. Alternatively, seropositivity for AQP4-Ab in two methodologically independent immunoassays is considered by some to be a valid substitute for NMO-IgG seropositivity (expert opinion).
